# Performance of an Expert Sensory Panel and Instrumental Measures for Assessing Eating Fruit Quality Attributes in a Pear Breeding Programme

**DOI:** 10.3390/foods12071426

**Published:** 2023-03-27

**Authors:** Lidia Lozano, Ignasi Iglesias, Jaume Puy, Gemma Echeverria

**Affiliations:** 1 Institute of Agriculture and Food Research and Technology (IRTA), Fruitcentre, Parc Agrobiotech, Parc de Gardeny, 25003 Lleida, Spain; 2 Agromillora Group, Plaça M. Raventós, 3, 08770 Sant Sadurní d’Anoia, Spain; 3 Department of Chemistry, University of Lleida, Rovira Roure 191, 25198 Lleida, Spain

**Keywords:** pear fruit quality, sensory descriptive analysis, instrumental measurements, expert panel

## Abstract

Breeding programmes count on stable trained panels that support breeding evaluation selections. This work aimed to evaluate the performance of a small expert panel in the join IRTA-PFR breeding programme to validate its use in the sensory assessments of fruit pear genotypes during the selection process. A breeding F1 population of 80 pear seedlings from this programme was used. Descriptors and standard references used for sensory evaluations of pear attributes were previously defined by the four members of the expert panel. A General Procrustes Analysis (GPA) was applied to analyse the relations between instrumental and sensory traits. The results showed a good relationship between sensory attributes such as firmness and crispness with penetrometer measures. A high correlation was also found between sensory sourness and titratable acidity (TA). Panel performance was evaluated in terms of reproducibility, homogeneity, and panel consonance. The results indicated that the experts were very consistent and had a good performance. The work demonstrates, for the first time, that a small expert trained panel could be efficiently used in pear breeding programmes and allows for the selection process in a more economical and available way in contrast to the larger sensory panels conventionally used.

## 1. Introduction

Pear (*Pyrus* spp.) is the second most globally important fruit tree crop within the Rosaceae family, just after apples (*Malus* spp.) and before peaches (*Prunus persica*). Pears are one of the most cultivated and grown fruit in temperate zones. China is the largest pear producer in the world (with almost 70% of production), while Spain is the third largest pear producing country in the E.U. [1], generating approximately 0.3 million tons per year [2], although production is limited to only a few well-known traditional varieties as ‘Blanquilla’, ‘Dr Jules Guyot’, and ‘Conference’. The same situation is given in other major European countries producing pears as Italy or Belgium on which the basis of the production is supported by two cultivars, ‘Abate Fetel’ and ‘Conference’. This evidences how difficult is to introduce and develop new pear cultivars in mature markets around the world. There are at least fifteen public and private pear breeding programmes in Europe that mainly focus on fruit quality or fruit type diversification [3]. A challenge for breeders is to develop new cultivars that capture the interest of pear consumers, for instance by improving either appreciated aspects of eating quality or offering different appearance fruit such as brilliant red or bicoloured ones. Both are common objectives of the main pear breeding programs currently developed [4]. At present, most fruit breeding programmes include sensory evaluations to identify and select new cultivars having high fruit quality that is likely to succeed in the market. Sensory evaluation judged by trained panels has been previously shown to be a reliable and consistent tool to investigate apple fruit quality in relation to breeding selection [5,6]. The main limitation of this procedure is the cost of the selection process to release new cultivars of superior eating quality.

New pear varieties must be of eating quality comparable or superior to those already existing, as well as having at least an adequate agronomical performance comparable with standard cultivars. Knowledge of individual sensorial characteristics of the fruit is therefore critical in understanding the eating quality of a new variety and its market potential. Usually, attributes such as soft and buttery flesh texture are considered important for high European pear quality, while crisp and cracking flesh are important for Asian pears [7]. Pear sensory attributes that are important to consumers have been studied by several authors. Juiciness, sweetness, acidity, aroma, astringency, aftertaste, flesh texture, and firmness are the most important sensory traits to be considered in pear fruit quality [8,9]. Gallardo et al. [10] determined the top-ranked quality attributes in pear as sweetness, juiciness, and firmness. Other studies indicated the ‘ideal pear’ for consumers only as sweet and juicy [11].

Although traditionally sensory analysis is done using large, trained panels [12], other studies on apples confirmed the validity of using smaller trained teams on sensory assessment for fruit breeding selection and postharvest evaluations in apples [13,14]. The cost/efficiency evaluation of descriptive analysis panels, specifically panel size, has been studied in various research papers [15,16,17]. These studies have evaluated the performance and sensory profiling of different panel sizes, ranging from full panels to smaller panels with 10 or fewer assessors. The results of these studies can help to determine the optimal panel size for a given sensory analysis project. However, as in this study, an expert panel is used, it is important to point out that even the expert panel counts with a lower number of assessors, experts are focused on the practical application of sensory attributes and can provide insight into the factors that influence the sensory quality [18]. In sensory analysis, panellists should be seen as an instrument for measuring sensory attributes. However, there are several problems related to the training, stability, and maintenance of the quality of these panels [19]. Training allows assessors to become familiar with vocabulary to improve discrimination ability and improve consensus within the panel. After training, the next step is to measure the degree of reliability by which evaluations are done [20]. 

Several statistical tools have been published to assist sensory professionals in evaluating panel and panellist performance, ranging from relatively simple statistics [21,22] and statistical models based on ANOVA [23] to multivariate models [24]. As such, descriptive sensory profiling is one of the most used sensory tools available to sensory professionals [25]. Despite the existence of different proposed methods to analyse sensory data, there is a consensus on the parameters to be considered for the evaluation of panels: reproducibility [21], homogeneity [26], and panel consonance [27]. These parameters can detect a lack of precision, disagreement, and ability or inability to discriminate between samples. The detection of these errors in a sensory panel is essential in assessing a product since the results of any descriptive profiling will be only as good as the performance of the panel [28]. On the other hand, statistical results of how a trained panel is working can be also directly related to interactions among sensory attributes. There is no work reporting both statements from pear sensory data.

Consumption of pears has steadily declined over the last 10 years [2]. Lack of flavour is one of the main reasons for the decrease in consumption. Consumers demand a better relationship between visual appearance, firmness, and organoleptic characteristics [29]. The improvement of fruit quality is the general objective of the IRTA-PFR pear breeding programme. In particular, the main goal is the finding new varieties with high good quality such as tasteful fruit with juicy flesh, but without a gritty texture that performs well under the hot dry climate of Northern Spain (or Catalonia) [30]. The selection process involves seedling fruit sampling at maturity and the sensory assessment of the fruit after storage. In this study, with the aim to provide results that are directly related to the eating experience of consumers, we designed a specific experiment to evaluate the performance of the expert panel that will carry out the sensory assessments of fruit pear genotypes in this selection process. Panel performance was evaluated in terms of reproducibility, homogeneity, and panel consonance. In addition, relationships between sensory attributes and instrumental measurements (fruit firmness, total soluble solids, and titratable acidity) were examined in order to have a better understanding of its correlation.

## 2. Materials and Methods

### 2.1. Plant Material

A breeding F1 pear population (80 seedlings) obtained from four controlled crosses made in 2002 in collaboration with Plant and Food Research (PFR, New Zealand) was used for the experiment. Crosses were made between PFR selections coming from inter-specific crosses between *Pyrus communis*, *P. x bretschneideri*, and *Pyrus pyrifolia*. The trial consisted of 80 trees, 20 trees per family, planted in a completely randomised four-block design in a research orchard of IRTA-Experimental Station Lleida placed at Gimenells (NE Spain). Trees were six-year-old, conducted in central axis, grown at 3.4 × 1 m spacing, and using standard commercial management practices recommended for the area, including fertiliser application and disease and pest control. Fruits were harvested once at optimum maturity (based on background colour changes and starch index) from each genotype and stored at 0–1 °C at 95% HR in air for 12 weeks. Fruits were removed from cold storage and were equilibrated at room temperature (20 °C) for 24 h before evaluation.

### 2.2. Instrumental Assessments

For each fruit (4 fruits per genotype, 80 genotypes, 320 fruits in total), firmness, total soluble solids (TSS), and titratable acidity (TA) were determined. Flesh firmness was measured as maximum force, after removing the skin on two opposite sides of each fruit, with a digital penetrometer (Model 53205; TR, Forlí, Italy) equipped with an 8 mm diameter plunger tip. TSS and TA were determined on cortical flesh juice extracted by an automatic juicer (Moulinex, Type BKA1) from one longitudinal half of each fruit. TSS was measured using a portable refractometer (Atago PR-32, Tokyo, Japan), and the results were expressed in °Brix. TA was measured with an automatic titrator Crison GLP 21 (Barcelona, Spain) and determined by titrating 10 mL of flesh juice with 0.1 N NaOH to pH 8.1 endpoint [31].

### 2.3. Sensory Analysis

#### 2.3.1. Sensory Panel

Four experts worked for four different sessions (one session per week), during which each expert evaluated 80 genotypes (20 genotypes along each session of 2.5 h). Experts were members of the IRTA-PFR pipfruit breeding team; they had years of experience in pipfruit sensory evaluations and had participated in several training exercises led by the sensory department of IRTA. Before the assessments of the present study, the experts had undertaken a week specific training course on pear sensory attributes (according to ISO 1993, no. 8586-1) to recognise attributes using specific evaluation techniques and to develop the standard references that will have to be used in the study. This course was followed by 1 h training sessions the day before each experiment assessment session to familiarise themselves with the intensities of the standard references and to evaluate a selection of test samples to provide feedback on the scoring of attributes to ensure consistency between the assessors. All panellists assessed all the genotypes. Each genotype was represented by four fruits which were divided into two replicates, each replicated of two fruits. Each expert assessed two fruits per tree (one of each replicate), and two experts independently scored a portion of the fruit they both shared. Therefore, each expert evaluated 40 samples per session (20 genotypes × 2 replicates). Within each session, the sample presentation was balanced for order, assessor, and replicate. This design provided eight measures of each sensory attribute per genotype (two per assessor per each of four fruit) for each of the 80 genotypes and the pairwise comparisons of the experts.

#### 2.3.2. Fruit Sample Preparation and Panel Test

Half of the fruit that was used in the sensory testing was further split into two halves with each portion being presented to a panellist for assessment. Thus, each panellist expert assessed one-quarter of the fruit. Peeled fruit samples were presented to the panellists in white plastic cups and were identified by a random 3-digit code. Samples were evaluated for sweetness, sourness, firmness, crispness, juiciness, grittiness, and astringency. The intensity of each of these seven attributes evaluated was qualified on a 10-point scale, with the lowest intensity corresponding to a score of 0 (none), while the highest intensity was scored as 9 (extreme/severe). Attribute definitions, techniques, and references are provided in Table 1. Panellists were instructed to use mineral water and crackers, which were provided as a palate cleanser between each sample assessment. Breaks of 10 min were allowed after evaluation of 20 samples. Data were collected on paper ballots. The sensory evaluation took place at the sensory department of IRTA under white illumination and at room temperature.

### 2.4. Statistical Analysis

Genotype average values were used to evaluate the correlation among sensory attributes and between sensory and instrumental data with the Proc CORR procedure of SAS 9.1 (SAS Institute, Cary, NC, USA). General Procrustes Analysis (GPA) was also performed with Senstools software v3.3 (Oliemans Punter and Partners, the Netherlands) to evaluate the correlation between sensory attributes and instrumental determinations. An improved consensus plot is generated in the GPA by allowing the data sets of each expert to be transformed by centring, rotating, and adjusting scales of each data set. Eigenvalue and variance for each dimension were computed using the Procrustes Analysis of Variance (PANOVA), which demonstrates the relative importance of each dimension of the model. A permutation test was run to estimate the significance of the GPA result. This test indicates a probability that the consensus derived from this study could have arisen by chance. 

To evaluate the performance of the panel, a two-way analysis of variance (ANOVA), in which experts, genotypes, and their interactions were considered, was carried out with the statistical software SAS 9.1 (SAS Institute, Cary, NC, USA). Additionally, to assess the ability of an assessor from a trained panel to differentiate samples, a one-way ANOVA was performed. Sensory data were analysed by the Simple Ranking test using the Friedman-type statistic test for rank data, with the non-parametric analogue to Fisher’s LSD for rank sums [32]. In that test, the null hypothesis of no sample difference at the α-level of significance is rejected if the value obtained exceeds the value of the test statistic T. If the X2-statistic is significant, the non-parametric analogue to Fisher’s LSD for rank sums is used. Principal component analysis was also applied to sensory data with XLSTAT software, version 2006.2 [33], to evaluate the consensus of the panel for each different attribute. Consonance values (C: the ratio between the first Eigenvalue and the sum of all the eigenvalues) were calculated for each attribute in order to determine the degree of consonance of the panel; a large C-value indicates that the assessors are consistent in their use of the attribute [27]. Table 2 summarises the tasks carried out and the statistical techniques that have been applied to each task.

## 3. Results and Discussion

### 3.1. Correlations among Measurements

Table 3 shows the Pearson’s correlation coefficients among instrumental and sensory attributes for all 80 pear genotypes. Across all 80 genotypes, moderate correlations were found between sweetness and the instrumental measures of TSS (r = 0.52, Table 3) and TA (r = −0.40), with the highest correlation being with the TSS/TA ratio (r = 0.58), as also reported by [8] in other pear cultivars. The penetrometer measurement was most highly correlated with sensory panellists’ ratings of firmness (r = 0.94) and crispness (r = 0.80,), an expected result since the instrumental puncture test has been associated as a predictor of sensory attributes for firmness and crispness in apples [34,35,36,37]. Juiciness was weakly correlated to penetrometer determination (r = −0.42), a similar association to what was found previously in pears [8]. According to De Belie et al. [38], there was a weak correlation between juiciness and penetrometer determination in pears. The study also found that the disparity in juiciness perceptions in apples and pears was due to differences in cell structure [37]. Significant differences were observed between the firmness levels of pears, instrumentally measured with the Sinclair iQ^TM^ system, and crispness, hardness, and fracturability [37].

In this study, a strong correlation was observed between firmness and crispness (r = 0.91); however, firmness was weakly and negatively correlated to juiciness (r = 0.30)—this result is in line with previous pear studies reported by Chauvin et al. [37] in apple and pear. A positive correlation was also found between sourness and astringency (r = 0.56), although this attribute has a very complex sensory perception [39]. The positive correlation between sourness and astringency in pears may imply that the sourer a pear is, the more astringent it will be. Astringency is generally considered a negative attribute in pears. However, sourness is an important sensory property of fruits that can affect their acceptability and consumption [40]. A close interaction with acidity has been previously reported in sensory science [28,41]. Grittiness was equally negatively correlated with firmness (r = −0.61) and crispness (r = −0.60). In general, the sweetness was weakly correlated with all other sensory traits, suggesting that sweetness perception in pear is affected by the inhibitory or masking interaction of other attributes [42].

### 3.2. GPA between Instrumental Measurements and Sensory Analysis

Relationships between instrumental and sensory data can also be studied by means of the Procrustes analysis [43]. GPA analysis mathematically transforms (scaling, translation, and rotation) the data and then corrects scale discrepancies among the different variables in the data sets [44]. 

**Figure 1:**
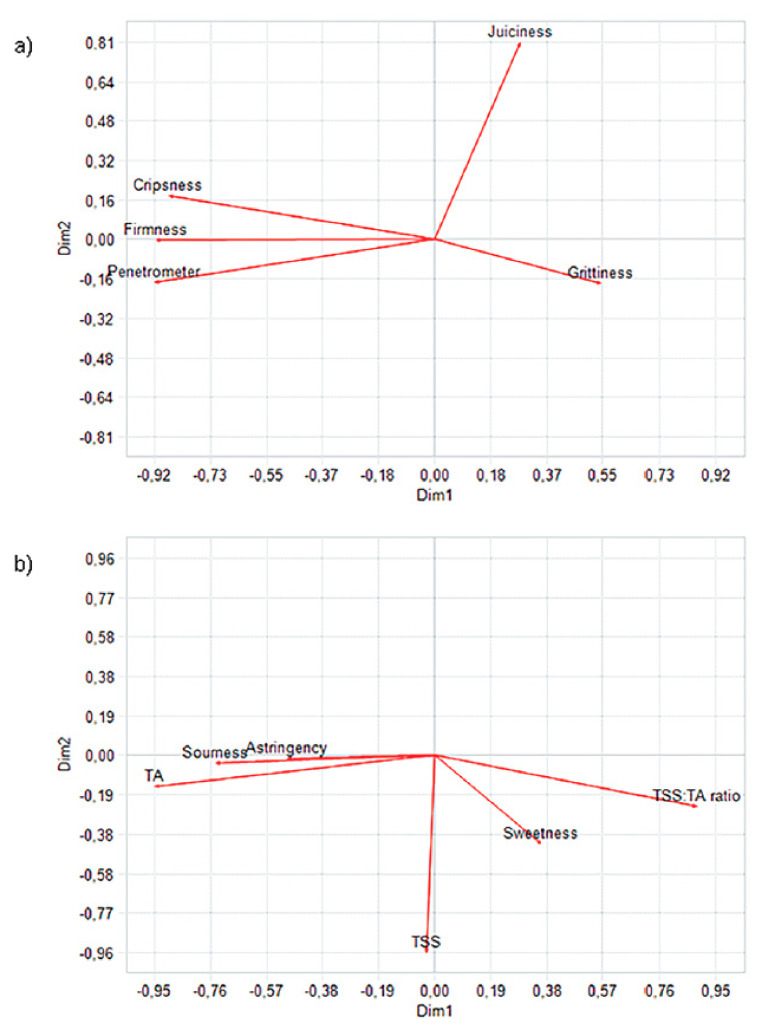
The consensus space of the first and the second dimension after GPA for all 80 pear genotypes: (**a**) relationship between texture sensory data (firmness, crispness, juiciness, and grittiness) and instrumental data (penetrometer); (**b**) relationship between flavour sensory data (sweetness, sourness, and astringency) and instrumental data (TSS, TA, and TSS/TA ratio).

The GPA model of the texture descriptors (sensory attributes firmness, crispness, juiciness and grittiness, and penetrometer measurements) for all 80 pear genotypes was explained by five dimensions. The first two dimensions extracted the 82.4% of the variance in the data, with the first dimension (Dim 1) explaining 70.0% and the second dimension (Dim 2) accounting for 12.4%. These values were well above the significant value for the upper 5% of the total variance accounted in the permutation test data set that could be achieved by chance as determined by the permutation test (37.11%) and indicating that true consensus was achieved (*p*< 0.05). To identify significant relationships between the attributes and dimensions, the correlation of the scores on the first three dimensions with the descriptors were considered (Table 4 and Table 5). These correlation coefficients with Dim 1 and Dim 2 are projections on the axis of the corresponding vector shown in Figure 1. The graphical examination showed a good agreement between firmness and crispness sensory vectors and instrumental firmness (penetrometer), which were aligned positively along the Dim 1 axis. Attribute juiciness was weakly associated with the penetrometer parameter (Figure 1a); this result is in agreement with previous work on pears [37], where a strong correlation among texture evaluation and sensory attributes of crispness and hardness was found, while juiciness of pear was weakly correlated to instrumental determination. Grittiness was correlated to the third dimension, although the percentage of variance explained for the third dimension was very low (3.84%). Moreover, the correlation scores were also low, and thus the relation between grittiness and the rest of parameters was insignificant. 

Figure 1b shows the consensus space of the first and the second dimension after GPA for all the 80 pear genotypes for the taste descriptors model (sweetness, sourness, and astringency, and TSS, TA, and TSS/TA). The first two dimensions of the GPA accounted for 92% of the variance (65% and 27% for Dim1 and Dim2, respectively), well above the significant value upper 5% of the total variance accounted in the permutation test data set (38.87%). Positive relationship along the Dim 1 axis was found between the sensory attribute sourness and TA. Sweetness vector was positively partially related along the Dim 2 axis with TSS and along the Dim 1 axis with the TSS/TA ratio—this result suggested that the sweetness perception of the fruit is affected by two aspects, the amount of TSS of the fruit and the TSS/TA ratio. In this way, taking two fruits with the same level of TSS, the sweetness perception of these fruits depended on the level of acidity that each fruit contained; therefore, at equal TSS content, one fruit with extreme TA (low TSS/TA ratio) was perceived as being less sweet. This fact is in agreement with Echeverría et al. [45], who showed that perception of sugar content can be masked or accentuated by the presence of acid. Astringency was related to the third dimension, given that the percentage of variance explained for the third dimension was low (7.45%), but the correlation score was moderate, and thus we could determine that astringency is slightly influencing the flavour perception. Astringency is caused by the formation of aggregated tannins and catechins in fruits such as pears, which can affect the quality of fruit flavour [39]. A negative correlation between astringency and sweetness perception was also observed in apples [46]. The influence of astringency on food flavour has also been reported by Canon et al. [47].

In this study, relationships between instrumental parameters with some sensory attributes in pear fruit (penetrometer measure with firmness and crispness and TA parameter with sourness) were found. However, the information extracted from instrumental analysis was not enough to evaluate other very important sensory attributes in pear quality such as juiciness and sweetness. These results agree with Harker et al. [34,48] in apples and Zerbini [29] in pears, who reported that the evaluation of texture properties, sweet taste, and flavour attributes require the assessment by trained sensory assessors, and thus, so far, physical or instrumental analysis cannot replace sensory analysis.

Given the good correlations observed between some sensory and instrumental measurements and given the need to evaluate many samples during the screening process, the use of these well-correlated instrumental measurements is proposed as a preliminary step. This will allow a first screening, by which undesirable fruit will be quickly eliminated and the number of samples to be evaluated by the expert panel will be reduced.

### 3.3. Panel Performance

#### 3.3.1. Reproducibility of Assessments of the Sample

Reproducibility is an important aspect of sample assessment and relates to the variability of the scores given to replicates of the same sample [49]. For proper assessment of reproducibility error in our study, the reproducibility of each assessor is compared with the reproducibility of the panel as a whole [22,50]. Therefore, the relative precision of the assessors in the evaluation has been estimated from each assessor’s contribution to the ANOVA’s total sum of squares of the error (SSE) for the whole panel [51]. 

Table 6 summarises, for each attribute, the result of a two-way analysis of variance in which experts, genotypes, and their interactions were considered, and the error variance was computed from the two replicates of each combination of expert–genotype.

Taking firmness as an example, an SSE (Sum of Square of the Error) of 205.12 was obtained (product of the error mean square, 0.64, and the degrees of freedom of the error, 320, as indicated in Table 6). The contribution of each expert panellist to the SSE of each attribute was calculated, and rank order numbers were assigned to the assessor. Following the example of firmness, the expert contributions to SSE were A = 56.50, B = 68.50, C = 43.50, and D = 36.50, and the rank numbers assigned were D = 1, C = 2, A = 3, and B = 4. In this way, a matrix of ordinal data with four rows (expert) and seven columns (attributes) was obtained, containing in each row the rank order number of the expert for the corresponding attribute. The obtained rank sums in increasing order (Table 7, second column) were analysed by the simple ranking test. The Friedman-type statistic for rank data was calculated (T = 1.80) and was compared to the critical value of X2 with three degrees of freedom. Accordingly, experts A, B, C, and D did not significantly differ between them (differences lower than 7.81 at the probability level of 0.05), which indicates that all experts had a good individual agreement with the panel as a whole. Similar results were obtained for the rest of the sensory attributes evaluated (Table 6). 

#### 3.3.2. Discriminatory Power of Each Expert

The power of discrimination of each expert was evaluated by a one way ANOVA analysis. The *F*-ratio associated with the F-test of the genotype effect on each expert was used to measure this discrimination (Table 8). Our results showed that all the experts were able to discriminate the genotypes on the basis of the considered attributes. For α < 0.01, the value of F_(79, 80)_ in the Fisher distribution table was 1.754. Comparing this value with our F ratio values for each expert and sensory attribute, we observed that all the experts obtained F ratio values higher than 1.754. These results mean that the variation among genotypes per expert was greater than we would expect to see by chance, indicating the high power of discrimination of our experts. 

#### 3.3.3. Homogeneity among Experts

Another important aspect of the panel performance is the homogeneity among experts in the evaluation of the same sample, which can be estimated from the interaction expert x genotype [26,51]. This analysis was carried out in a similar procedure to that mentioned above for reproducibility by considering the individual contribution of each expert to the interaction expert x samples (SSI). 

Taking again firmness as an example, a total SSI = 279.1 was calculated as the product of F value of the interaction effect x error mean square x degrees of freedom of the interaction (Table 6). Expert contribution to each attribute was computed, and rank orders were assigned to the panellists according to their increasing contribution to SSI. A matrix of ordinal data was obtained and analysed as above. The T value for rank data was calculated (T = 2.49) and was compared to the critical value of X2 with three degrees of freedom. As a result, experts A, B, C, and D did not significantly differ (differences in rank were lower than 7.81 at the probability level of 0.05). The same was observed for the rest of the evaluated sensory attributes (Table 6).

#### 3.3.4. Panel Consonance

**Figure 2:**
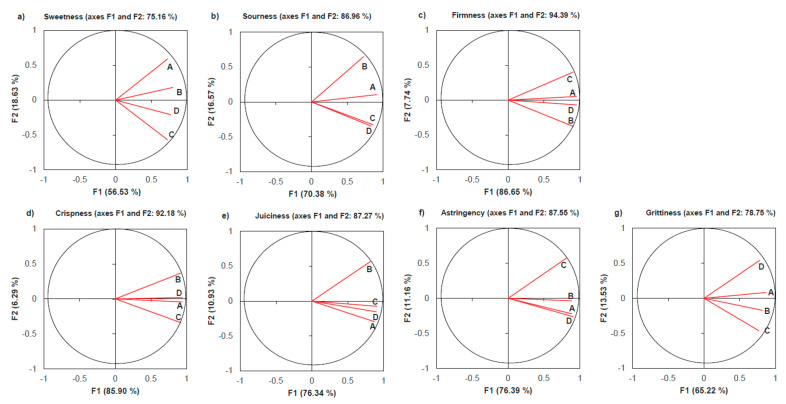
Loading plot F1 versus F2 of the principal components analysis models of each sensory attribute, (**a**) for sweetness, (**b**) for sourness, (**c**) for firmness, (**d**) for crispness, (**e**) for juiciness, (**f**) for astringency and (**g**) for grittiness, with the samples characterised by the scores of each expert panellist (four experts: A, B, C, D; samples *n* = 80).

Consonant analysis was performed to evaluate the level of agreement within the panel according to the method proposed by Dijksterhuis [27]. For each attribute, a PCA was carried out on the matrix of between assessors’ correlation calculated from the matrix of attribute scores, made by the genotypes (rows) and experts (columns). The loading plot of the PCA models of each attribute with the percentage of variance explained by the first two principal components (F1 and F2) are shown in Figure 2.

While PCA provides a graphic illustration of how individual panellists used the attributes to describe the samples [28], the consonance coefficients (C) (ratio between the first Eigenvalue and the sum of the others) were calculated to provide a numerical indicator to the agreement between judges in using the attributes [27] (Table 9). High ratios indicate one-dimensional solutions, or in other words, that the panellists used the attribute in a similar way, in which case the explained variance on the first principal component of each attribute can be interpreted as a percentage of panel agreement. 

Two attributes (firmness and crispness) showed the highest coefficients of consonance with the second Eigenvalue, clearly lower than the first one (Table 9). In this way, the best agreement among panellists was observed for these two attributes with 86.65% (firmness) and 85.90% (crispness) of total variance explained by the first PC (Figure 2). Three other attributes (juiciness, sourness, and astringency) showed intermediate coefficients of consonance, with the first Eigenvalue being roughly twice that of the second one. This result can indicate a slightly bi-dimensional solution. In Figure 2, expert B can be identified as responsible for the deviation from unidimensionality in the evaluation of juiciness and sourness, while expert C was outlying for astringency. Both assessors will need further calibration training for these particular attributes. The remaining two attributes (sweetness and grittiness) showed the lowest coefficients of consonance, and a lower percentage of variance explained for the first principal component, 56.53% for sweetness and 65.22% for grittiness. Overall, the results of the consonance analysis suggest a generally good agreement among experts, although further training in the use of some attributes may further improve the results. Similar results in sourness were reported by Echeverría et al. [52] when evaluating the consistency of a training panel of apples. 

## 4. Conclusions

In relation to the correlation between sensory attributes and instrumental measurements of texture and flavour, we observed a positive correlation between the instrumental measurement of firmness and some sensory attributes of texture. The results of the evaluation of the pear expert panel in the present study (reproducibility, homogeneity, and panel consonance) indicate that the experts were very consistent and indicated a good panel performance. Accordingly, the sensory assessments of fruit pear genotypes made by this four-member expert panel can be used with strong confidence in the pear breeding selection process. Our results evidence that a small panel of experts with a good agreement among them could provide a good assessment when selecting fruit pear genotypes. This work demonstrates for the first time in pear selection that the methodology exposed could be efficiently used in other deciduous species because of its good reproducibility, homogeneity, and consonance. In addition, this small panel allowed us to conduct the selection in an easier and cheaper way compared to an expert panel, which is increasingly more important to measure the efficiency of the currently running breeding programs.

## Figures and Tables

**Table 1:** Descriptors and standard references used for sensory evaluations of pear attributes.

Attribute	Definition	Reference Standard	Intensity on 10-Point Scale
Sweet taste	Characteristic of sugar	50% juice *	4
		Pear juice	9
Acid taste	Characteristic of acid	Pear juice	4
		Yogurt	9
Astringency	Drying of the oral tissue	Pomegranate	3
		Quince	9
Crispness	The amount and pitch of sound when the sample is first bitten with the front teeth	Banana	0
		Celery	9
Firmness	The force required to bite through the sample	Avocado	0
		Carrot	9
Juiciness	The amount of juice released by the sample when chewing with the back teeth	Avocado	0
		Watermelon	9
Grittiness	The presence of small hard particles in the flesh	Avocado	0
		‘Limonera’ pear	6
		Ground rice	9

* 50% Juice = Pear Juice/Granini^®®^) diluted 50/50 with filtered water (all solutions refer to 1 L).

**Table 2:** Performed task, techniques, and procedures.

Task	Statistical Technique	Procedure
Relation among variables	Pearson’s correlation	Estimated by REML method
Sensory–instrumental relationship	General Procrustes Analysis	Attributes with absolute correlation higher that 0.5 with one of the first dimensions
Panel performance		
Reproducibility	Two-way ANOVA and Simple Ranking test	The contribution of each panellist to the ANOVA sum of squares of the error (SSE) was obtained for each attribute and panellists were ordered according to increasing contributions
Homogeneity	Two-way ANOVA and Simple Ranking test	The contribution of each panellist to the ANOVA sum of squares of the interaction panellist x sample (SSI) was obtained for each attribute and panellists were ordered according to increasing contributions
Consonance	Principal component analysis (PCA) with data from each individual attribute	The ratio between the first Eigenvalue and the sum of the others were obtained from PCA results for each attribute and use as coefficient of concordance

**Table 3:** Pearson’s correlation coefficients among instrumental and sensory attributes. Correlation coefficients were calculated on mean value per genotype.

	Sweetness	Sourness	Firmness	Crispness	Juiciness	Astringency	Grittiness	P	TSS	TA	TSS/TA
Sensory attributes											
Sweetness	1.00	−0.32 **	−0.33 **	−0.28 *	0.40 **	−0.42 ***	0.32 **	−0.34 **	0.52 ***	−0.40 **	0.58 ***
Sourness		1.00	ns	ns	0.27 *	0.56 ***	ns	ns	ns	0.85 ***	−0.73 ***
Firmness			1.00	0.91 ***	0.30 *	ns	−0.61 ***	0.94 ***	ns	ns	ns
Crispness				1.00	ns	ns	−0.60 ***	0.80 ***	ns	ns	ns
Juiciness					1.00	ns	ns	−0.42 ***	ns	ns	ns
Astringency						1.00	ns	ns	ns	0.44 ***	−0.43 ***
Grittiness							1.00	−0.63 ***	ns	ns	ns
Instrumental measures											
Penetrometer (P, Kg)								1.00	ns	ns	ns
Total soluble solids (TSS, °Brix)									1.00	ns	ns
Titratable acidity (TA, % acid malic)										1.00	−0.79 ***
Ratio TSS/TA											1.00

Significant levels are indicated by asterisks: * *p* < 0.05, ** *p* < 0.005, *** *p* < 0.0001, ns = no significant (*p* > 0.05).

**Table 4:** Correlation coefficient (*r* > 0.5) between the texture descriptors and the first three dimensions.

Dimension 1		Dimension 2		Dimension 3
Positive Axis	Negative Axis	Positive Axis	Negative Axis	Positive Axis
	Firmness (−0.91)	Juiciness (0.80)		Grittiness (0.55)
	Crispness (−0.87)			
	Penetrometer (−0.92)			

**Table 5:** Correlation coefficient (*r* > 0.5) between the taste descriptors and the first three dimensions.

Dimension 1		Dimension 2		Dimension 3
Positive Axis	Negative Axis	Positive Axis	Negative Axis	Positive Axis
TSS/TA ratio (0.88)	Sourness (−0.74)		Sweetness (−0.43)	Astringency (0.71)
	TA (−0.95)		TSS (−0.96)	

TSS: total soluble solids, TA: titratable acidity.

**Table 6:** Two-way analysis of variance of seven sensory attributes carried out for 4 expert panellists on 80 pear genotypes.

Attribute	F Values			Error
	Expert (E)	Genotype(G)	Ex G	Mean Square
Sweetness	18.26	7.01	1.92	0.50
Acidity	42.71	11.88	1.78	0.57
Firmness	39.50	35.02	1.84	0.64
Crispness	24.35	34.04	1.90	0.68
Juiciness	21.13	12.93	1.35	0.80
Astringency	49.43	12.53	1.44	0.82
Grittiness	70.28	7.79	1.47	0.82
Degrees of freedom	3	79	237	320

**Table 7:** Results of the Simple Ranking test applied to ordinal data matrices obtained by assigning rank order number to experts according to their (a) contributions to the sum of squares of the error (SSE) and (b) contribution to the sum of squares of the interaction expert x sample (SSI).

Expert	SSE	SSI
D	15	14
A	16	16
C	18	19
B	21	21

Minimum significant difference between rank sums, 9.27 (*p* = 0.05).

**Table 8:** Discrimination power of experts as assessed by the F ratio of the F test.

	Sweetness	Sourness	Astringency	Crispness	Firmness	Juiciness	Graininess
**Expert 1**	3.3711	7.2377	4.3356	10.056	8.5044	3.9002	4.7008
**Expert 2**	2.9732	5.0872	4.1296	10.1086	8.6105	3.6197	3.2082
**Expert 3**	3.1678	3.519	2.8357	8.8049	11.0147	4.1345	1.8922
**Expert 4**	3.3384	2.8964	4.7737	10.9344	14.2173	5.7448	2.1331

**Table 9:** Panel consonance. Results from independent PCA of the data for each attribute obtained from the Conventional Descriptive Profile.

Attributes	Coefficient of Consonance (C)	Ratio between the First and the Second EIGENVALUES
Firmness	6.49	11.20
Crispness	6.09	13.66
Astringency	3.24	6.84
Juiciness	3.23	6.99
Sourness	2.38	4.25
Grittiness	1.88	4.82
Sweetness	1.30	3.03

## Data Availability

The data presented in this study are available on request from the corresponding author.
